# Development of a pharmaceutical science systematic review process using a semi‐automated machine learning tool: Intravenous drug compatibility in the neonatal intensive care setting

**DOI:** 10.1002/prp2.1170

**Published:** 2024-01-11

**Authors:** D. Thisuri N. De Silva, Brioni R. Moore, Tobias Strunk, Michael Petrovski, Vanessa Varis, Kevin Chai, Leo Ng, Kevin T. Batty

**Affiliations:** ^1^ Curtin Medical School Curtin University Perth Western Australia Australia; ^2^ Curtin Health Innovation Research Institute Curtin University Perth Western Australia Australia; ^3^ Medical School The University of Western Australia Crawley Western Australia Australia; ^4^ Wesfarmers Centre for Vaccines and Infectious Diseases Telethon Kids Institute Nedlands Western Australia Australia; ^5^ Neonatal Directorate King Edward Memorial Hospital, Child and Adolescent Health Service Subiaco Western Australia Australia; ^6^ Pharmacy Department, King Edward Memorial Hospital Women and Newborn Health Service Subiaco Western Australia Australia; ^7^ University Library, Curtin University Perth Western Australia Australia; ^8^ School of Population Health Curtin University Perth Western Australia Australia; ^9^ Curtin School of Allied Health Curtin University Perth Western Australia Australia; ^10^ School of Health Sciences Swinburne University of Technology Hawthorn Victoria Australia

**Keywords:** machine learning, pharmaceutical science, physicochemical compatibility, systematic review

## Abstract

Our objective was to establish and test a machine learning‐based screening process that would be applicable to systematic reviews in pharmaceutical sciences. We used the SPIDER (Sample, Phenomenon of Interest, Design, Evaluation, Research type) model, a broad search strategy, and a machine learning tool (Research Screener) to identify relevant references related to y‐site compatibility of 95 intravenous drugs used in neonatal intensive care settings. Two independent reviewers conducted pilot studies, including manual screening and evaluation of Research Screener, and used the kappa‐coefficient for inter‐reviewer reliability. After initial deduplication of the search strategy results, 27 597 references were available for screening. Research Screener excluded 1735 references, including 451 duplicate titles and 1269 reports with no abstract/title, which were manually screened. The remainder (25 862) were subject to the machine learning screening process. All eligible articles for the systematic review were extracted from <10% of the references available for screening. Moderate inter‐reviewer reliability was achieved, with kappa‐coefficient ≥0.75. Overall, 324 references were subject to full‐text reading and 118 were deemed relevant for the systematic review. Our study showed that a broad search strategy to optimize the literature captured for systematic reviews can be efficiently screened by the semi‐automated machine learning tool, Research Screener.

## Graphical abstract

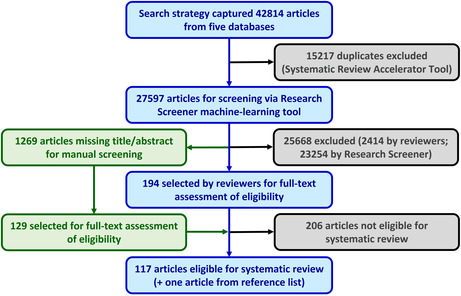

Overview of systematic review search, screening and selection process.

**Abbreviations**
NICU: neonatal intensive care unit
PICO: Population, Intervention, Comparison, Outcomes
PRISMA: Preferred Reporting Items for Systematic Reviews and Meta‐Analyses
SPIDER: Sample Phenomenon of Interest, Design, Evaluation, Research type

## 1 INTRODUCTION

Well‐conducted systematic reviews and meta‐analyses are considered to provide the highest level of evidence for informed decisions in policy and practice. The process for systematic reviews is typically defined by well‐established models, such as PICO (Population, Intervention, Comparison, Outcomes)^1^ and SPIDER (Sample, Phenomenon of Interest, Design, Evaluation, Research type).^2^ PICO is commonly used for systematic reviews of clinical research, whereas SPIDER appears to offer advantages for other scientific disciplines.

The required methodological rigor of systematic reviews is associated with significant time and economic demands,^3^ with screening of titles and abstracts considered to be the most time and labor‐intensive component of the review process.^4^ Hence, there is a growing interest for more automated solutions to facilitate systematic reviews.^5^ However, despite the profusion of systemic reviews in recent years, there is a paucity of reviews in the pharmaceutical sciences disciplines which have used the SPIDER model and evaluated a machine learning screening tool to expedite the process.

The introduction of new technologies, such as web‐based tools, for streamlining the screening process has provided promising results by substantially reducing the time for initial screening. Machine learning‐based screening tools include Rayyan,^6^ Abstrackr,^7^ RobotAnalyst,^8^ and ASReview.^9^ Nevertheless, there are limitations and barriers to the widespread use of some screening tools, including the risk of missing articles (this could be improved by semi‐automation), a requirement to “train” the program by initially screening a high number of articles (limiting the time savings), the use of a dedicated computer/server for installation of the screening tool, or failure to adapt to multiple platforms.^4^ Research Screener (RS), a semi‐automated machine learning tool, has the advantage of applying contemporary Natural Language Processing algorithms and is able to train itself for abstract ranking from a small selection of seed abstracts.^4^ By contrast, other tools such as Rayyan may require numerous seed abstracts for training its model.^10^ Research Screener also has practical advantages; for example, it can be used on a wide range of hardware platforms and it does not require a dedicated server.^4^

**FIGURE 1:**
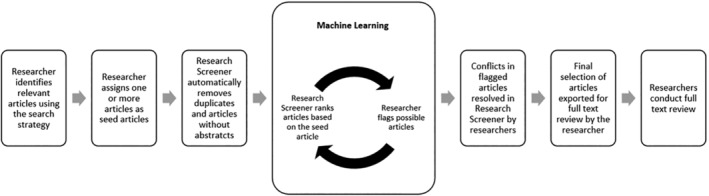
Research Screener assisted screening process (adapted from Chai et al.^4^).

The Research Screener process is illustrated in Figure 1. Two items are initially provided to Research Screener by the researchers as separate files from the reference manager software: (i) All potentially eligible articles retrieved from the systematic review search strategy and (ii) at least one seed article assessed as highly relevant. Using the seed article(s) abstract, the Research Screener algorithm ranks articles by relevance and the screening process commences with presentation of the top 50 unread articles (cycle 1). Independently, members of the review team screen the abstracts of these 50 articles to flag those which are deemed relevant according to predetermined inclusion criteria for the systematic review. The titles are retained for full article screening and, in conjunction with the irrelevant (discarded) articles, are used to refine the Research Screener algorithm. Research Screener re‐ranks articles in the set of records (references) available for screening to determine the next 50 most relevant articles (cycle 2) and the process continues in cycles of 50 articles. The screening process ceases when either all articles have been screened by the reviewers or the research team completes screening to a level of confidence that all relevant articles have been identified (e.g., several cycles with no article selected as relevant). Upon completion of the initial screen, the principal reviewer can access the combined results, including conflicts in the flagged articles (i.e., disagreements between the individual reviewers). The conflicts are resolved in Research Screener, by an open process of consideration by the reviewers and/or an independent third reviewer. The final selected articles (flagged by both reviewers and the resolved conflicts) are exported for full‐text review.

We report the process of establishing and testing a robust literature search strategy in accordance with the SPIDER model and the use of Research Screener^4^ in the reference selection process for a systematic review in pharmaceutical sciences. The aim of our systematic review was to collate the current evidence on intravenous drug compatibility as applicable to y‐site administration in neonatal intensive care (NICU) settings. To the best of our knowledge, no systematic reviews have been conducted to evaluate peer‐reviewed physicochemical compatibility studies in this context. Two systematic reviews with related objectives (drug compatibility in adult intensive care settings) have been reported previously^11^, ^12^ and were conducted by manual screening of up to 2000 citations.^11^

## 2 METHODS

### 2.1 Development of the search strategy

The research question, “In‐vitro studies conducted to evaluate the physical and chemical compatibility of intravenous drugs used in NICUs,” was defined in consultation with members of the research team (TDS, BRM, TS, MP, KTB). The SPIDER model (Sample, Phenomenon of Interest, Design, Evaluation, Research type) for systematic reviews^2^ was adapted for the protocol of the present review, which was registered in Open Science Framework (https://doi.org/10.17605/OSF.IO/XGK6V). The search strategy (Table 1) was structured as three concepts (categories), the first of which focused on compatibility, incompatibility, and stability terms. The second concept focused on intravenous, injection, and y‐site terms, and the third comprised a list of drugs based on expert panel review (TS, MP, KTB) of a compilation of neonatal drug protocols from seven health‐care institutions (four different countries; TDS).

**TABLE 1:** Final search strategy for the systematic review of intravenous drug compatibility in the neonatal intensive care setting.

Concept 1	Concept 2	Concept 3
compatib*	intravenous*	NICU drugs
incompatib*	intra‐venous*	(6 drugs in pilot study)
stability	iv	(95 drugs in full review)
instability	y‐site	
	y‐ site	
	ysite	
	injection*	
	infusion*	
	parenteral	
	injectable*	
	mixture*	

The search concepts were pilot tested (TDS, VV) in iterative stages, using the Embase database and various terms within concepts 1 and 2, and a panel of six drugs (aminophylline, indometacin, ketamine, pentoxifylline, caffeine, and sotalol). The six drugs were selected on the basis of their potential relevance to the planned systematic review and a total known list of 59 articles, which was determined from a standard reference source^13^ and our own independent, manual literature search. The optimum search strategy captured 1622 articles and included all known articles of interest.

The first stage of evaluating the screening process was to test the feasibility and reliability of title reading only. Two independent reviewers (TDS, KTB) manually screened a random selection of 400 titles from the set of 1622 references (25%) and the kappa coefficient^14^ was calculated to determine the inter‐reviewer reliability associated with title reading as a screening process for the systematic review.

As Research Screener had not previously been used in a pharmaceutical sciences systematic review, the full set of 1622 articles was then used to pilot test the tool. Three seed articles were used and two reviewers (TDS, KTB) conducted the screening process, with the kappa coefficient calculated to assess the inter‐reviewer reliability.

### 2.2 Application of search strategy and Research Screener tool

Based on the pilot study results, the search strategy was applied to all 95 drugs in concept 3 (Table 1) and five databases, comprising two inter‐disciplinary (Proquest and Web of Science) and three intra‐disciplinary databases (Embase, Medline, and Cinahl) to retrieve articles. The retrieved references were initially deduplicated using a validated deduplication tool “Systematic Review Accelerator” and the final library was entered into Research Screener. Eight articles were identified to provide seed abstracts for the screening process. Following exclusion of articles by Research Screener (comprising conference proceedings, duplicates, and articles with no abstracts), the reviewers proceeded with independent cyclical screening of the captured articles. The reviewers also manually screened (by title) the articles with no abstracts and the kappa coefficient was determined to quantify reviewer agreement for each relevant process.

## 3 RESULTS

### 3.1 Manual screening versus semi‐automated screening (Research Screener)

The kappa coefficient from the manual screening pilot study of 400 titles was 0.75, which suggests “moderate agreement”^14^ in the reviewers' title screening process. In the pilot study using Research Screener, 98 references (out of 1622) were removed because they did not contain abstracts (e.g., letters, editorials, and short communications, because abstracts are essential for the Research Screener machine learning cycles). These excluded titles were separately exported back to the reference manager software and saved in a separate group for manual screening. The remainder (1524) were directed for screening by Research Screener (TDS, KTB). Fifteen conflicts were subsequently resolved by the reviewers. The kappa coefficient following screening of the full set of pilot study articles via Research Screener was 0.86, which was indicative of “strong” agreement between the two reviewers.^14^

### 3.2 Main review

**FIGURE 2:**
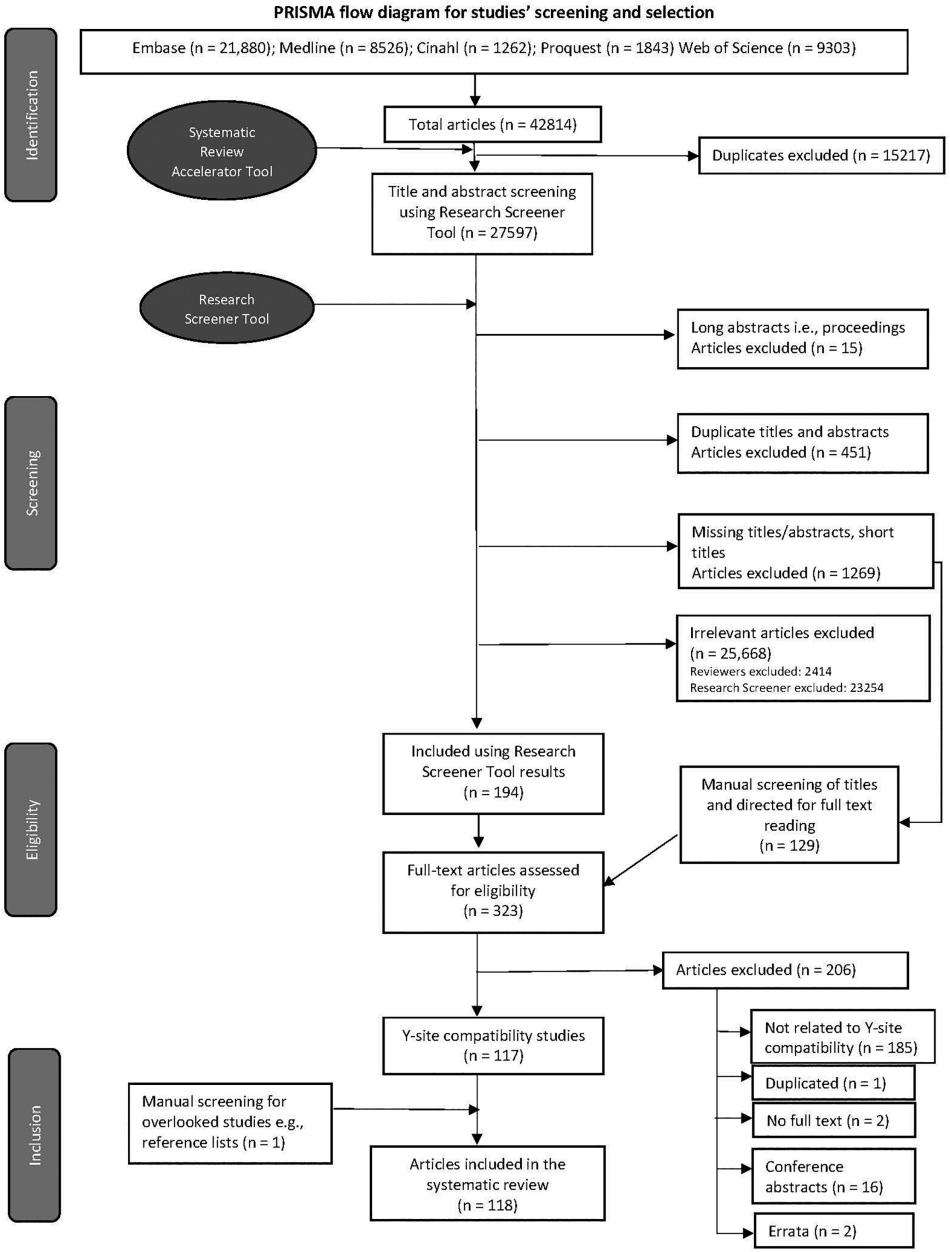
PRISMA* flow diagram for the systematic review search, screening, and selection process (*PRISMA: Preferred Reporting Items for Systematic Reviews and Meta‐Analyses).

A total of 42 814 results were retrieved from the selected databases (Embase—21 880, Medline—8526, Cinahl—1262, Proquest—1843, Web of Science—9303) and the Systematic Review Accelerator deduplication process retained 27 597 references for further screening (Figure 2).

Research Screener initially removed 15 long abstract articles (i.e., conference proceedings in which the reference manager record contains all conference abstracts combined), 451 duplicated titles/abstracts, and 1269 articles with missing abstracts/titles, from the full set of 27 597 records (Figure 2). The 1269 articles with no abstract/title included short reports, editorials, letters, and notes, and were directed for manual screening by the reviewers. The remainder (25 862) were subject to screening by the two independent reviewers in cycles of 50, as outlined above.

**FIGURE 3:**
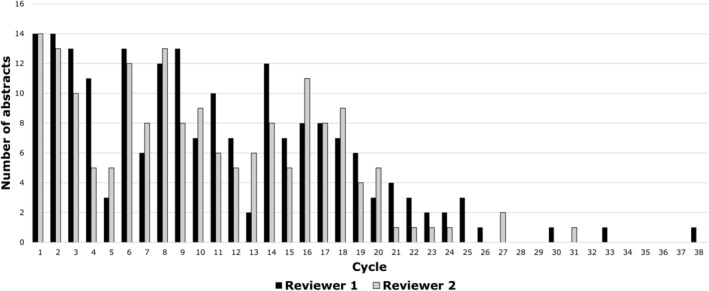
Number of abstracts flagged by each reviewer for full‐text review in the Research Screener process. Reviewers 1 and 2 completed 52 and 35 cycles, respectively.

Reviewer 1 completed 52 cycles of screening via Research Screener, which comprised 10% of the references available for screening and concluded after 14 cycles with no abstracts selected (Figure 3). Reviewer 2 completed 35 cycles (6%) of screening and concluded after four cycles with no abstracts selected. As a result, 149 articles were flagged by both reviewers. A further 67 were selected by only one reviewer and classified as conflicts for resolution by the review team, from which 37 were considered potentially eligible and included in the full‐text review. Including the eight seed abstracts, a total of 194 articles (0.75%) were directed for full‐text consideration at this stage. The kappa coefficient was 0.80, indicating strong agreement.

The 1269 references without titles/abstracts were screened manually by the two reviewers to select potentially eligible reports for full‐text read (most included a title and were only missing an abstract) and 129 were selected for progression to full‐text review (kappa coefficient 0.78, indicating moderate agreement).

Overall, a total of 323 articles were subject to full‐text reading, of which 117 were found to fully comply with the inclusion and exclusion criteria and were included in the formal systematic review (reported elsewhere). Screening of reference lists of the selected articles identified one further study which was not captured in the initial search strategy and was therefore included in the final total of 118 articles for systematic review (Figure 2).

**FIGURE 4:**
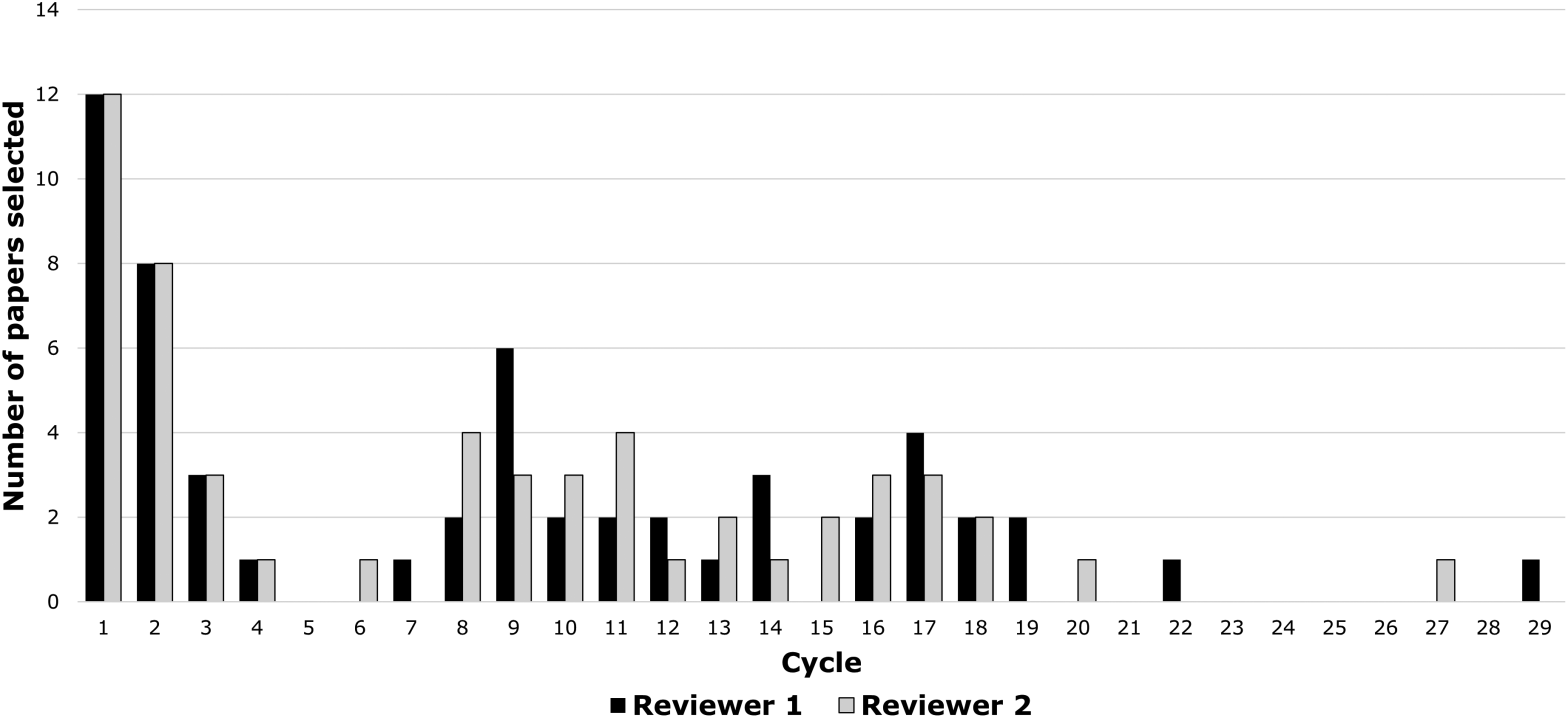
Number of papers selected for the review (after full‐text read) from each screening cycle.

Further insights to the value of Research Screener are shown in Figures 3 and 4. Of the 186 articles which were directed to full‐text read (excluding the eight seed abstracts), 55 were eventually selected for inclusion in the systematic review. Reviewer 1 encountered all 55 articles by the 29th cycle of article flagging (1408 papers, 5.4%) and reviewer 2 by the 27th cycle (1304 papers; 5%). Similar results were observed in an acute pain systematic review, where all of the reviewed articles were identified after screening 5% of the search results.^4^

The cyclical trends in selection of studies for the systematic review (Figure 4) demonstrate that Research Screener presented 44% (24/55) of the articles to the reviewers in the first four cycles, ostensibly due to the effective use of the eight seed abstracts. Thereafter, selection rates varied between the two reviewers and became sporadic after 19 cycles.

In order to estimate the potential time saved by completing the screening process from <10% of the full search strategy results, screening time data for each reviewer were extracted from Research Screener and analyzed. The mean (95% confidence interval; range) time to screen each title/abstract in the final 20% of cycles screened by the two reviewers was 8.4 (6.8–10.1; 2–131) and 15.2 (12.6–17.9; 1–244) seconds, respectively. The final 20% of cycles was selected for this analysis because it represented a continuous series of cycles in which relatively few papers were potentially eligible, thus providing a plausible, conservative estimate of the time to screen subsequent cycles, if this had been required. Therefore, based on the >23 250 titles/abstracts that did not require screening, the potential time saving was at least 56 and 98 h for each reviewer.

## 4 DISCUSSION

Our study has demonstrated the combined use of the SPIDER systematic review model, a broad search strategy to capture over 27 000 deduplicated articles and screening via the machine learning tool, Research Screener, to expedite the extraction of eligible articles for a pharmaceutical science systematic review. We tested the literature search and screening process using a pilot study and assessment of inter‐reviewer reliability.

In the process of establishing the final search strategy, we found the large number of captured articles was unavoidable, since our endeavors in the pilot study to constrain the search had excluded essential references. It became apparent that our search strategy required several generic terms, such as “stability,” “compatib*,” “intravenous*,” and “injection*” (Table 1), and we concluded this requirement to include common terms may be a broader issue for systematic reviews in pharmaceutical sciences and other scientific disciplines. Hence, the iterative process of the pilot study was an important evaluation step in developing our systematic review, to maximize the capture of relevant references, and we would encourage this course of action. The value of machine learning screening tools is that large databases from search strategies can be efficiently managed to extract articles for full‐text review.

The pilot study indicated that 7.3% (119/1622) of the captured articles could be relevant to our systematic review, which was comparable to 7.5% in a previous study,^11^ and therefore suggested approximately 2000 articles would be identified as potentially eligible for the systematic review. However, the proportion of articles selected for full‐text review was lower than predicted from the pilot study and appeared to be related to at least two factors. Firstly, many of the selected articles included several drugs from concept 3 of the search strategy (Table 1), thus limiting the overall pool of eligible studies. Second, in retrospect, the pilot study included some intravenous drugs which are more commonly used in neonatal/pediatric settings than in adult patients, or for which there is a limited body of relevant, published literature (e.g., caffeine, pentoxifylline, indomethacin, and sotalol). The reviewers noted (anecdotally) that some terms, such as stability and intravenous, are used in a wide range of contexts and a number of abstracts were easily and swiftly excluded. Importantly, due to the machine learning algorithms and user‐friendly operation of Research Screener, the overall workload impact in the screening process was modest. Further investigation of the reasons behind the relatively low selection rate from the initial pool of articles was outside the scope and value of the present study, as the goal was to optimize capture of eligible papers.

There was an appreciable time saving associated with Research Screener. Recent reports indicate the time to screen abstracts for systematic reviews ranges from 30 to 60 s per abstract and varies according to the experience of the reviewer.^4^, ^7^, ^15^, ^16^ In the present study, the two reviewers noted that screening the cycles with a rich source of eligible papers was more time consuming than the latter cycles (after cycle 20), where most abstracts could be rapidly excluded. As a result of the Research Screener ranking and screening process, whereby the average title/abstract screening time from the final 20% of cycles for the two reviewers was 8.4 and 15.2 s, respectively, the overall time saving was at least 56 and 98 h, respectively, if screening the results of the full search strategy was necessary.

One limitation of Research Screener and similar tools is the preclusion of papers which do not contain an abstract. In our systematic review, the reviewers were required to manually screen 1269 such references; however, there was moderate inter‐reviewer agreement, and this was an important pool of articles in the present study, contributing approximately half of the final body of literature for the systematic review.

Overall, we have shown the importance of testing the systematic review search strategy process and optimizing the literature captured. Semi‐automated machine learning tools such as Research Screener may then be utilized to efficiently screen the results of the search strategy, providing a manageable workload and confidence in the outcomes and scientific rigor of the systematic review.

## AUTHOR CONTRIBUTIONS

KTB, BRM, and TDS conceived the study, with advice from TS and MP. All authors contributed to the study design. TDS and KTB had principal responsibility for acquiring the data; BRM was the independent monitor and VV contributed to the pilot study. KTB and TDS conducted initial analysis and interpretation of the data, with advice from all authors. KTB and TDS prepared the first draft of the manuscript. Revision and additional contributions to the manuscript were provided by all authors. All authors approved the final manuscript.

## CONFLICT OF INTEREST STATEMENT

At the time of submission, KC and LN are the developers of the Research Screener software and receive financial remuneration to maintain the hosting platform and its related requirements. All other authors declare no conflicts of interest.

## 5 Ethics statement

Not applicable.

## Data Availability

Data not provided in the manuscript are available on reasonable request to the authors.
